# Glucocorticoid Treatment in Acute Respiratory Distress Syndrome: An Overview on Mechanistic Insights and Clinical Benefit

**DOI:** 10.3390/ijms241512138

**Published:** 2023-07-28

**Authors:** Jinquan Zhang, Peng Ge, Jie Liu, Yalan Luo, Haoya Guo, Guixin Zhang, Caiming Xu, Hailong Chen

**Affiliations:** 1 Department of General Surgery, The First Affiliated Hospital of Dalian Medical University, Dalian 116011, China; 2 Institute (College) of Integrative Medicine, Dalian Medical University, Dalian 116044, China; 3 Laboratory of Integrative Medicine, The First Affiliated Hospital of Dalian Medical University, Dalian 116011, China; 4 Department of Molecular Diagnostics and Experimental Therapeutics, Beckman Research Institute of City of Hope, Biomedical Research Center, Comprehensive Cancer Center, Monrovia, CA 91016, USA

**Keywords:** glucocorticoid, severe acute pancreatitis, acute lung injury, acute respiratory distress syndrome, mechanism, sepsis

## Abstract

Acute lung injury/acute respiratory distress syndrome (ALI/ARDS), triggered by various pathogenic factors inside and outside the lungs, leads to diffuse lung injury and can result in respiratory failure and death, which are typical clinical critical emergencies. Severe acute pancreatitis (SAP), which has a poor clinical prognosis, is one of the most common diseases that induces ARDS. When SAP causes the body to produce a storm of inflammatory factors and even causes sepsis, clinicians will face a two-way choice between anti-inflammatory and anti-infection objectives while considering the damaged intestinal barrier and respiratory failure, which undoubtedly increases the difficulty of the diagnosis and treatment of SAP-ALI/ARDS. For a long time, many studies have been devoted to applying glucocorticoids (GCs) to control the inflammatory response and prevent and treat sepsis and ALI/ARDS. However, the specific mechanism is not precise, the clinical efficacy is uneven, and the corresponding side effects are endless. This review discusses the mechanism of action, current clinical application status, effectiveness assessment, and side effects of GCs in the treatment of ALI/ARDS (especially the subtype caused by SAP).

## 1. Introduction

Glucocorticoids (GCs) are “stress hormones” that affect the metabolic processes of almost every cell type. Under relaxed conditions, healthy individuals secrete about 20 mg GC/day but, under stress, secretion can reach 300 mg/day [1]. The circadian rhythm also regulates GCs production, which reaches its highest level in the blood during the early morning and drops to its lowest point at nightfall. This secretion pattern helps to regulate chemokine and inflammatory cytokine production and to moderate metabolism [2]. Each cell type has a distinct response to GCs and changes in GC structure and concentration affect the human body in different ways [3]. Meanwhile, GC function is dependent on its specific molecular structure, and GCs’ clinical efficacy is most dependent on the degree and duration of GC exposure to the GC receptor (GR). Maximal GRα saturation induced by different drug doses, start times, and mode and duration of administration ensures that GC efficacy is optimized [4]. In a randomized controlled trial of 17 ICU patients with moderate-to-severe ARDS, the administration of 20 mg/day intravenous GCs for 5 days followed by 10 mg/day from day 6 onwards downregulated inflammation, increased tissue repair, and elevated the alveolar-capillary membrane permeability index, reducing mechanical ventilation duration and overall mortality [5]. In recent decades, GCs have been increasingly used to treat several conditions, including various autoimmune diseases, rheumatoid arthritis, acute lung injury/acute respiratory distress syndrome (ALI/ARDS), sepsis, allergic skin diseases, and the suppression of transplant graft rejection [6]. GCs have also been shown to have a therapeutic effect against coronavirus disease 2019 (COVID-19). In a 2020 study of 173 patients with severe COVID-19 pneumonia from 14 distinct respiratory high-dependency units (RHDUs), early and extended administration of low-dose methylprednisolone (MP), a synthetic GC, improved the systemic inflammatory response, increased oxygenation markers, and reduced invasive mechanical ventilation and mortality rates [7].

Severe acute pancreatitis (SAP), which mediates the secretion of several inflammatory mediators and leads to ALI/ARDS, is a common clinical disease. ALI is the most important cause of the early death of SAP patients. Xuan et al. [8] surveyed 45 hospital ICUs and found that, while routine GC use remains controversial, most physicians still chose a short-course and low-dose GC treatment early in the disease process. However, several reports of adverse side effects have been recorded. GCs are often effective at improving clinical symptoms but rarely cure the disease. Thus, before prescribing GCs, it is important to define the corresponding parameters needed to decide whether to continue treatment [9].

Several studies have assessed the underlying mechanisms of SAP-induced SIRS and potential treatments for this condition. However, the mechanism of GC-mediated SAP-ALI tissue repair has not been reviewed to date. The current article discusses the pathogenesis and clinical management of SAP, with particular emphasis on the role of GCs. The review begins with a comprehensive overview of GCs, including their chemical structure, tissue distribution, and the downstream GR. The GCs and GR types associated with SAP are summarized and their anti-inflammatory mechanisms and immunomodulatory roles in regulating SIRS and ALI/ARDS are introduced. The article concludes with a summary of the use of GCs for SAP treatment along with their associated side effects and contraindications. The findings presented here can be used to inform future studies and aid the development of effective SAP treatments.

## 2. Overview of Glucocorticoids

GCs are used to treat SAP patients with SIRS because of their potent anti-inflammatory effect and extensive therapeutic activity. GCs reduce monocyte-macrophage levels in the lungs and lower the production of inflammatory mediators. While effective against SAP, GCs can cause a range of side effects. As a result, clinicians and scientists are particularly focused on how GCs can be administered safely and effectively. This section provides an overview of the sources, structures, and modes of action of endogenous GCs, such as cortisone and cortisol, and exogenous GCs, such as prednisone, prednisolone, methylprednisolone, and dexamethasone, while emphasizing the current state of their use as a treatment for SAP.

### 2.1. Endogenous GCs

#### 2.1.1. GC Definition, Source, and Production

Endogenous GCs refer to endogenous steroid hormones generated in the body; their primary source is the zona fasciculata of the adrenal cortex, where steroidogenic enzymes in the mitochondria catalyze their synthesis. In addition, a number of tissues, including the brain, vasculature, thymus gland, and epithelial barriers, produce small amounts of extra-adrenal endogenous GCs. However, the amount of endogenous GCs in the systemic circulation is extremely low, and they exert primarily localized physiological effects. Whether produced within or outside of the adrenal gland, endogenous GCs are essential for the regulation of numerous physiological functions in the human body [9]. Exposure to physical (trauma, cold), chemical (strong acid, strong alkali), or biological (bacterial, viral infection) stimuli induces neuronal regulation of the hypothalamus–pituitary–adrenal axis. This causes the release of GCs that serve to control metabolism and maintain the stability of the body’s external and internal environments. While effectively keeping the individual alive, this stress response can also have adverse effects on the body [3].

**Figure 1:**
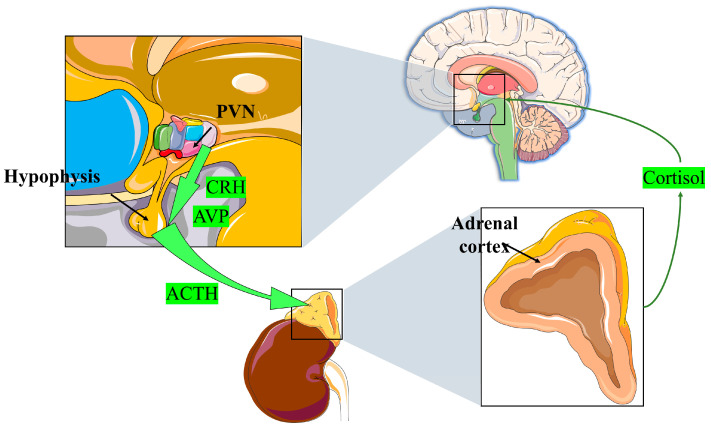
Hypothalamic–pituitary–adrenal axis. PVN: hypothalamic paraventricular nucleus; CRH: corticotrophin-releasing hormone; AVP: arginine vasopressin; ACTH: adrenocorticotrophic hormone.

The central nervous system responds to environmental stimuli by releasing corticotrophin-releasing hormone (CRH) and arginine vasopressin (AVP) (Figure 1). CRH and AVP further regulate the secretion of adrenocorticotrophic hormone (ACTH) from the anterior pituitary, ultimately leading to GC release from the zona fasciculate of the adrenal cortex, a process known as “hypothalamic–pituitary–adrenal (HPA) axis” signaling [10,11]. This is the primary mechanism for GC production in the physiological state. Other tissues in the body, including the brain, thymus, vasculature, and intestinal epithelium, can also produce GCs but the effects of these sources on GC concentrations in vivo are negligible [12,13,14,15,16].

A high level of GCs, in turn, can prevent the hypothalamus and pituitary gland from reducing CRH and ACTH production. Both genomic and non-genomic mechanisms are involved in this negative feedback regulatory system. First, excess GCs can directly inhibit CRH-R1 and POMC gene expression, thereby inhibiting CRH secretion in the hypothalamus. Second, GCs can directly act on CRH neurons and either inhibit the release of endocannabinoids or trigger the release of the inhibitory neurotransmitter, gamma-aminobutyric acid (GABA). This negative feedback mechanism helps to stabilize endocrine function [9]. Ectopic ACTH syndrome and adrenal sebaceous adenoma are not affected by this control mechanism. Thus, tumor cells release GCs autonomously, and the negative feedback process remains unbalanced, resulting in paraneoplastic, Meador, and Carney syndromes. These endocrine disorders can have adverse effects on the body [17].

#### 2.1.2. GCs Structure

**Figure 2:**
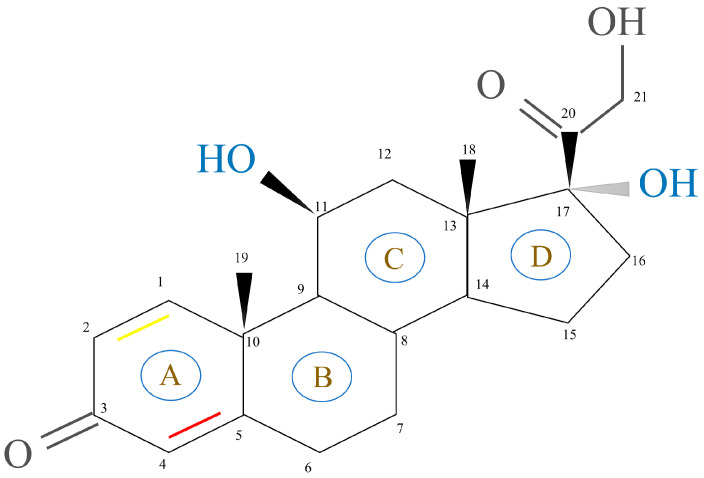
Structure of the steroid nucleus. The glucocorticoid activity depends on the double bond between carbon 4 and 5 (red bond) in ring A, but the double bond between carbon 1 and 2 (yellow bond) in the same ring can enhance the glucocorticoid activity selectively.

GCs, aldosterone (MC), and dehydro-epi-androsterone (DHEA) are secreted by the zona fasciculate, zona glomerulosa, and zona reticularis of the adrenal cortex, respectively. This process is catalyzed by steroidogenic enzymes in the mitochondria of different synthetic cells and leads to the production of cholesterol [18]. The basic structure of steroidal hormones is the steroid nucleus (Figure 2). Fluorination of the 1,2 double bond of the A ring and the 6α and 9α positions of the B ring can induce GC activity. This, in turn, is dependent on the substitution of the C ring with a 11β hydroxyl group. Most synthetic GC drug modifications are based on this carbon skeleton [19]. The changes in GC molecular conformations are reflected by changes in both their biological activity and affinity to cortical-binding globulin and cells. These findings indicate how dependent GCs’ clinical efficacy and function are on their structure [3].

#### 2.1.3. GCs Mechanisms of Action

GCs impact many physiological functions including growth and development [20], metabolism [21], water and electrolyte balance [22], mood and cognitive function [23,24], immune responses, and inflammatory reactions [25,26]. All of these processes are dependent on the interaction between GCs and nuclear receptors (NRs) such as the progesterone receptor (PR), estrogen receptor (ER), androgen receptor (AR), and mineralocorticoid receptor (MR) [27]. When GCs binds to GR, the zona fasciculata induces the secretion of GCs into the bloodstream. Plasma proteins bind to GCs and serve as transporters. About 80–90% of GCs are coupled to corticosteroid-binding globulin (CBG), while the remaining 10% are linked to albumin and remain inactive [28]. If infection or other external stimuli occur during the transport process, CBG is hydrolyzed by elastase, an enzyme produced by neutrophils, which triggers the release of bound GCs [29]. Free GCs diffuse through the cell membrane to perform their functions and remain in a balanced state of active and inactive forms in the cytoplasm [30]. GR is mostly an intracellular receptor present in almost all cell types. Once GCs bind to GR, an activated receptor complex is formed. This complex entity connects to the relevant gene region and controls the production of glucocorticoid response elements (GREs) farther down the signaling pathway [10,31]. In stress conditions, endogenous GCs can also play a pro-inflammatory role [25,32].

### 2.2. Exogenous GCs

Because of their significant anti-inflammatory and immunosuppressive properties, exogenous GCs, a type of exogenous/synthetic steroid hormones, are widely employed for the treatment of numerous inflammatory and autoimmune illnesses [33]. Exogenous GCs have a long history, dating back to 1930, when animal adrenal cortex extracts were utilized as an alternative treatment for individuals suffering from adrenal insufficiency. Synthetic cortisone acetate was approved for use in the treatment of Addison’s disease in 1948. Exogenous GCs, such as prednisone and prednisolone, were discovered and used clinically in the 1960s [34,35]. The number of exogenous GCs available and the ways they can be administered have both grown in recent years to accommodate a wide range of clinical applications [36].

Few studies have assessed the clinical role of GCs in the prevention of AP complicated by ALI/ARDS, and there are still no clear guidelines on the optimal dose and timing and duration of use. Exogenous GC drugs can be short-, medium-, and long-acting and the duration of action is short, medium, or long term. In the clinic, the most common short-acting preparations are hydrocortisone and cortisone, the most used intermediate-acting preparations are prednisone, prednisolone, and methylprednisolone (MP), and the most prescribed long-acting preparation is dexamethasone (DEX) [37,38,39,40,41]. The structure of natural endogenous GCs, cortisol and hydrocortisone, served as the basis for the development of these manufactured exogenous GCs [19]. Compared with endogenous GCs, synthetic GCs have improved bioavailability, potency, and GR binding specificity. In addition, some synthetic GCs, including DEX, have better stability and cannot be inactivated by 11β-hydroxysteroid dehydrogenase (11β-HSD) 2 [42,43,44].

One of the most essential enzymes involved in GC and mineralocorticoid (MC) function is 11-β-hydroxysteroid dehydrogenase (11β-HSD). 11β-HSD 1 is a reductase that is mostly found in liver tissue and is responsible for turning inactive GCs into active GCs. 11β-HSD 2 is mostly found in the target tissues, including the salivary glands, kidneys, and colon, of different MCs, and is responsible for oxidizing them. Enzyme action converts active GCs into inactive GCs [44].

#### 2.2.1. Short-Acting GCs

**Figure 3:**
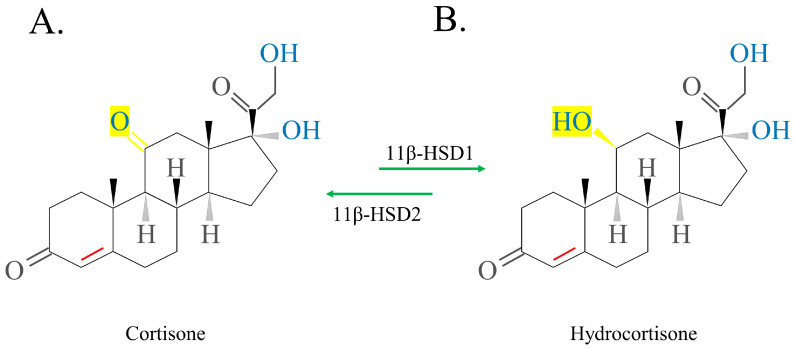
Structure of cortisone and hydrocortisone. The double bond between carbon 4 and 5 (red bond) in ring A is crucial for the glucocorticoid activity. The enzyme 11β-hydroxysteroid dehydrogenase 1 (11β-HSD1) converts the inactive cortisone to the active hydrocortisone, while the enzyme 11β-hydroxysteroid dehydrogenase 2 (11β-HSD2) reverses this process.

The schematic structure of exogenous cortisol/hydrocortisone, which can be extracted from the adrenal cortex of animals, is shown in Figure 3. Activated hydrocortisone (HC) (Figure 3B) is activated by inactive cortisone (Figure 3A), promoting 11β-HSD1 to replace the 11-ketol on the C ring with a 11-hydroxyl radical. Deactivation occurs in the opposite manner. The conversion of exogenous cortisone to HC occurs primarily in the liver. Thus, in patients with abnormal liver function, activated cortisone is preferred over inactivated cortisone [3,9,34].

#### 2.2.2. Medium-Acting GCs

**Figure 4:**
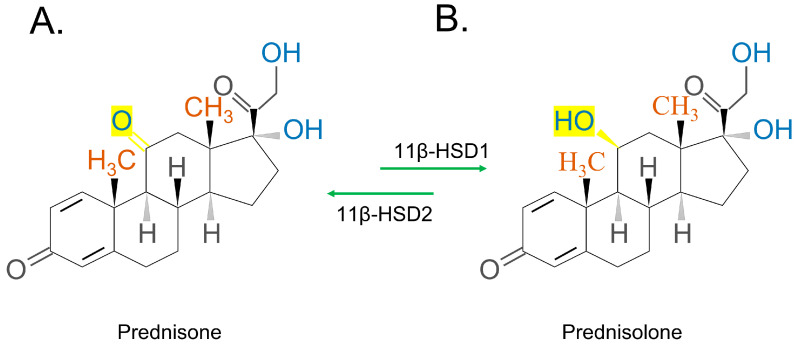
Structure of prednisone and prednisolone. The enzyme 11β-HSD1 converts the inactive prednisone to the active prednisolone, while the enzyme 11β-HSD2 reverses this process.

The chemical structures of the synthetic GC steroids, prednisone and prednisolone, are 11b,17a,21-trihydroxy-pregna-1,4-diene-3,20-dion and 17a,21-dihydroxy-pregna-1,4-diene-3,11,20-trione, respectively [35]. Prednisone (Figure 4A)/prednisolone (Figure 4B) has a similar conversion process as cortisone/hydrocortisone, which is also mediated by 11β-HSD (Figure 4). Inactive prednisone or dihydrocortisone are converted to active prednisolone or dehydrocortisol, through reduction by 11β-HSD 1. The opposite process occurs when 11β-HSD 2 oxidizes prednisolone/dehydrocortisol [44,45].

**Figure 5:**
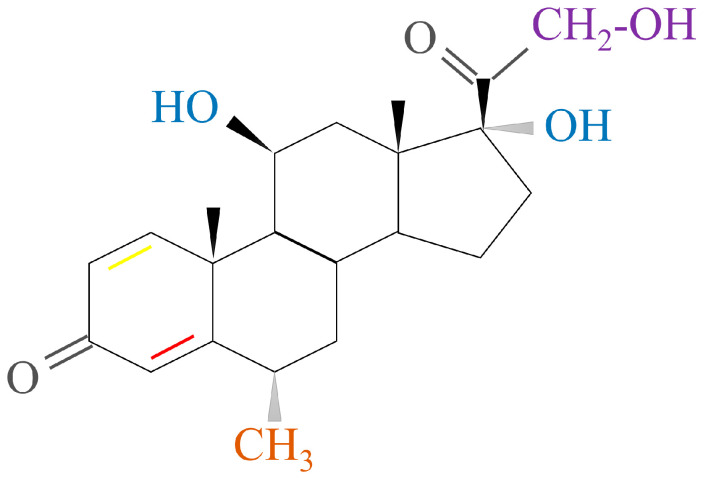
Structure of methylprednisolone. The glucocorticoid activity depends on the double bond between carbon 4 and 5 (red bond) in ring A, but the double bond between carbon 1 and 2 (yellow bond) in the same ring can enhance the glucocorticoid activity selectively.

MP, a synthetic glucocorticoid steroid, has four times the anti-inflammatory strength of HC and is also longer lasting (plasma half-life: HC = 1.5 h, MP = 2.5 h) [46]. The chemical structure of MP is shown in Figure 5.

#### 2.2.3. Long-Acting GCs

**Figure 6:**
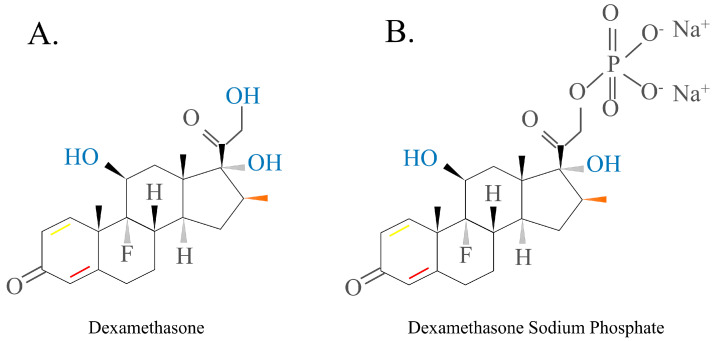
Structure of dexamethasone and dexamethasone sodium phosphate. The red bond in ring A is essential for glucocorticoid activity, while the yellow bond can selectively increase it. DEX is converted within the organism into the active form, the water-soluble form DEX sodium phosphate (**B**), which has low lipid membrane permeability and is used as a more soluble form of the active pharmaceutical ingredient in intravenous drugs. DEX sodium phosphate can be converted by hydrolysis reactions to DEX and sodium phosphate.

DEX, a fluorinated HC analogue, has the formula 9-fluoro-11β,17,21-trihydroxy-16α-methylpregna-1,4-diene-3, and 20-dione (Figure 6A). Since DEX contains fluorine atoms, it has ~75 times more pharmacological activity than prednisolone and approximately 35 times more activity than cortisone. DEX’s duration of action is 36–72 h [6,45,47].

Dexamethasone sodium phosphate is the DEX form that is most commonly used in the clinic because it has reduced lipid membrane permeability and is more soluble in water (Figure 6B). This drug is often used as an active pharmaceutical ingredient in intravenous drugs because its anti-inflammatory, anti-allergic, and immune-regulating functions mimic those of natural GCs [48,49,50,51].

## 3. GCs Mechanism in SAP-SIRS-ALI Treatment

### 3.1. Core Pathology of SAP-SIRS-ALI

Acute pancreatitis (AP) is a potential complication of abdominal surgery. Serious AP can lead to ALI/ARDS and is a common reason for AP-associated mortality. Currently, no treatment exists to slow progression. As a result of population growth, environmental changes (including changes in dietary patterns and increasing obesity and smoking rates), and improvements in diagnostic techniques, the annual global incidence of AP is increasing. In 2019, there were 34 cases of AP (95% CI: 23–49) per 100,000 in the general population and 1.16 deaths (95% CI: 0.85–1.58) per 100,000 in the general population [52]. This common and complex disease is also putting a major burden on national health care costs. AP-related admission costs in the United States were USD 2.2 billion in 2003 (95% CI: 2.0–2.3 billion), with a mean cost per hospital day of USD 1670 (95% CI: 1620–1720), and an average cost per hospital stay of >USD 10,000. These findings underscore the importance of identifying cost-effective treatment strategies and illustrate that the early identification and prevention of disease progression can significantly reduce hospital costs [53].

The mechanisms involved in the pathological process of SAP causing ALI/ARDS have been outlined by our research team in a previous review. Damage to acinar cells is triggered by abnormal activation of pancreatic trypsin induced by various etiologies including glandular pathological changes such as calcium overload of alveolar cells and the disruption of pancreatic microcirculation. This promotes the destruction of mechanical, chemical, biological, and immune barriers in the intestine. Accompanied by inflammatory cell necrosis and the high production of inflammatory mediators such as IL-1β and TNF-α, this inflammatory storm increases local inflammation to SIRS, indicating that AP has begun the early stage of multi-organ dysfunction syndrome (MODS). After extensive damage to alveolar epithelial cells, capillary endothelial cells, and the blood–brain barrier caused by elevated inflammatory cell numbers and cytokine production, ALI/ARDS begins to develop [54].

**Figure 7:**
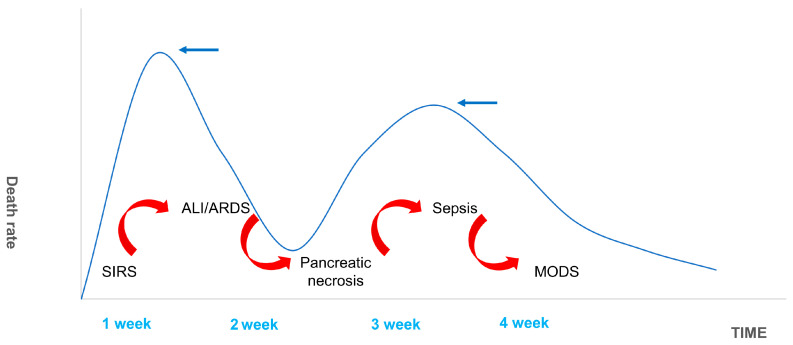
Death Spikes of SAP. The figure shows the distribution of deaths in SAP patients. The first peak of death occurred in the first week or so after SAP, and respiratory failure caused by ALI/ARDS complicated by SIRS was the most common. The second death peak was due to sepsis secondary to MODS caused by pancreatic necrosis infection. It is usually located in the third and fourth weeks.

The 1992 American College of Chest Physicians/Society of Critical Care Medicine (SCCM) Consensus Conference Committee defines this as an uncontrolled host response to infectious or non-infectious injury [55,56]. Excessive cytokine production disrupts the balance of pro- and anti-inflammatory cytokines and signals the development of SIRS. The inflammatory mediators involved in this pathological process include tumor necrosis factor-α (TNF-α), interleukin-1β (IL-1β), interleukin-6 (IL-6), interleukin-10 (IL-10), intercellular adhesion molecule-1 (ICAM-1), platelet-activating factor (PAF), CD40L, complement component C5a, substance P, hydrogen sulfide (H2S), and chemokines [57,58,59,60,61]. SIRS is a double-edged sword for the body. While fighting pathogens or the body’s own necrotic tissue, SIRS can have many harmful effects including altered immune status, organ dysfunction, and a disruption in the procoagulant/anticoagulant balance [62]. There are two mortality peaks associated with SAP (Figure 7). The initial peak occurs 1–2 weeks following the onset of symptoms. MODS, which is induced by SIRS, is the most common cause of death at this time point, accounting for 60–80% of all fatalities. The second peak occurs approximately 2 months after onset. Most of these deaths are caused by sepsis and other infections, and the length of SIRS is directly associated with the severity of SAP and its prognosis [63]. Hietaranta et al. [55] showed that the risk of systemic complications increases as the severity of SIRS rises.

During the early stages of SAP, pro-inflammatory processes destroy pathogens and dead tissue but may also cause irreversible damage to the host. At the same time, the compensatory anti-inflammatory response syndrome (CARS) works to limit damage to host tissue without interfering in pathogen clearance [64]. CARS was proposed in 1996 by Bone [65], who served as the chair of the ACCP/SCCM Consensus Conference. In contrast to SIRS, CARS restores balance from the inflammatory state through systemic inactivation of the immune system. It is important to note that CARS and SIRS can occur concurrently [64]. Gunjaca et al. [66] showed that SIRS and CARS are pathogenic states associated with the early acute inflammation response to AP and their intensities are correlated. This study used the SIRS/CARS model to measure AP severity. There are several possible ways in which this immunological response may occur. When SAP is initiated, there is a high production of pro-inflammatory mediators and a low production of anti-inflammatory mediators. During the early stages of the disease, mixed anti-inflammatory response syndrome (MARS) occurs and high amounts of pro- and anti-inflammatory mediators are produced. In the end stage, CARS, associated with high levels of anti-inflammatory mediators and few or no pro-inflammatory mediators, occurs [67]. Thus, maintaining the balance between pro- and anti-inflammatory cytokines early in SAP and alleviating any tissue and organ damage is critical to preventing MODS and reducing mortality [68,69].

### 3.2. Research Progress of GC in SAP-SIRS-ALI

GCs are the most used drug for the treatment of ALI/ARDS caused by SAP and can effectively reduce patient mortality. After binding to GR and moving into the nucleus, GCs play a significant role in promoting an anti-inflammatory response, reducing oxygen-free radical damage, and improving microcirculation [70].

Kimura et al. [71] found that, following adrenalectomy, AP rats were more sensitive to acinar cell apoptosis due to reduced endogenous cortisol production. These animals had greater pancreatic edema, higher amylase levels, more potent inflammatory responses, and higher rates of mortality. These effects were significantly reduced following exogenous GC administration [72]. These studies provide evidence that GCs reduce the onset of AP [73]. The following sections explore the roles of GCs in each pathological mechanism of SAP-SIRS-ALI.

#### 3.2.1. Glucocorticoid Receptor (GR)

Before discussing GC functions, it is important to provide an overview of GR, a member of the NR superfamily [74]. GR is a ligand-dependent nuclear receptor (NR) that belongs to the nuclear receptor superfamily’s subgroup 3, which also includes steroid receptors, the progesterone receptor (PR), mineralocorticoid receptor (MR), estrogen receptors (ERα and ERβ), and androgen receptor (AR). GR is an important mediator of GCs’ physiological and pharmacological actions. GR binds to ligands and can regulate downstream gene transcription via two major molecular mechanisms: first, it binds directly to GC response elements (GREs) on DNA to activate or inhibit target gene expression; second, it binds to other transcriptional components and can activate or inhibit target gene expression. These genes play a role in a variety of physiological processes, including inflammation, development, metabolism, and stress [75,76].

**Figure 8:**
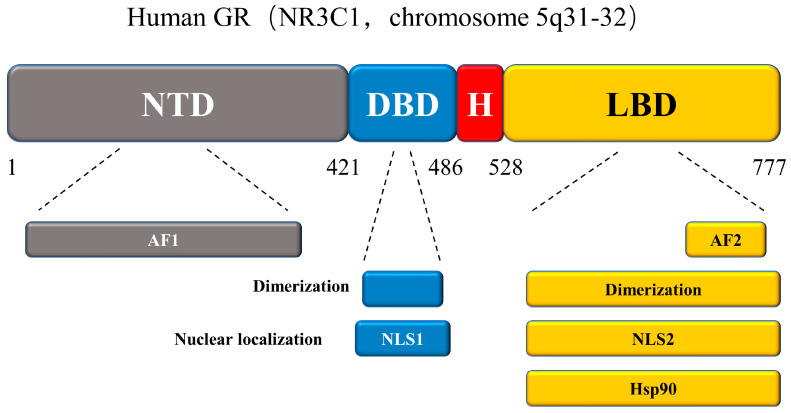
The structure of GR protein. NTD: N-terminal transactivation domain; DBD: DNA-binding domain; H: Hinge region; LBD: the C-terminal ligand-binding domain; AF1: activation function-1; AF2: activation function-2; NLS1 and NLS2: nuclear localization signals. An N-terminal domain, a disulfide-bond-forming domain (DBD) with two zinc fingers, a hinge region, and a C-terminal LBD make up the GR protein structure. GR’s many structural domains have an effect on its function. Below, we label the primary functional domains of the GR protein, changes to which may result in dysfunction.

New GR isoforms with distinct expression patterns, properties, and types of gene regulation are continuously defined. These isoforms are produced from a single gene but are made more complex and diverse through alternate splicing and translation initiation processes. After a series of translational modifications, GR functional diversity is further expanded. Here we discuss the structure of the GR protein, which contains four functional domains (Figure 8): an N-terminal transactivation domain (NTD), a central DNA-binding domain (DBD), a C-terminal ligand-binding domain (LBD), and a hinge region [76,77]. The NTD structure of GR contains post-translational modifications (PTMs) and is responsible for transcriptional activation. As the main element of the transcriptional activation function, activation function-1 (AF-1) engages with key components of the transcription machinery and coregulators and is a significant location for PTMs. Of these, the DBD is the most conserved. In addition to its role in DNA binding, GR dimerization, and nuclear translocation, the DBD recognizes and binds to the target DNA sequences with GREs. The hinge region of this protein serves as a pliable connection between the DBD and the LBD. The LBD connected to the hinge region is composed of 12 α-helices and 4 β-sheets, forming a pocket-like structure that binds to and co-regulates with GCs in a ligand-dependent manner. The LBD contains transcriptional activation function-2 (AF2) and a GR domain that binds to Hsp90 [78,79,80,81].

**Figure 9:**
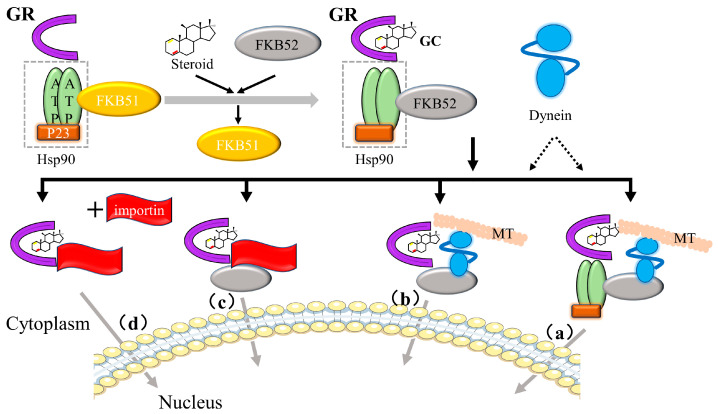
The process of GCs and GR transport into the nucleus. MT: microtubules; Normally, GR-Hsp90 first binds to the immunophilin FKBP51. After encountering GCs, FKBP52 replaces FKBP51 and interacts with dynein to link the GR-Hsp90 complex to microtubules for transport to the nucleus. The composition of the transport complex that binds to GC/GR during this transport has not been fully elucidated yet, and the following four modalities may exist: (a) the transport complex contains Hsp90, microtubules, dynein, and FKBP52; (b) the transport complex contains microtubules, dynein, and FKBP52; (c) the transport complex contains FKBP52 and importin; and (d) the transport complex contains only importin.

Before binding to GCs, GR binds to the chaperone proteins p23, hsp70, and hsp90, and immunophilins, FKBP51 and FKBP52, of the FK506 family to form a large multi-protein complex that exists in the cytoplasm to maintain their transcriptionally inactive but high-affinity ligand-binding conformation [82,83]. GC binding causes the conformation of GR to change and the large multi-protein complex to dissociate, exposing NLS1 and NLS2 nuclear localization signals (placed at the DBD/hinge region junction and LBD region, respectively) and allowing the complex to rapidly pass through the nuclear pore (Figure 9) [76].

**Figure 10:**
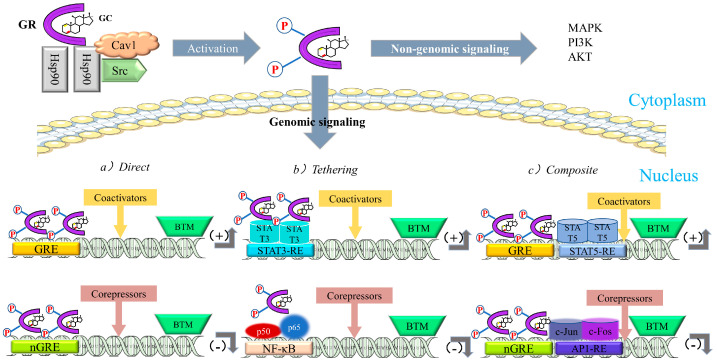
The regulatory pathway of GR. Cav−1: caveolin−1; Src: steroid receptor coactivator; STAT: signal transducer and activator of transcription; BTM: Basal transcription machinery; GR regulates gene expression through three mechanisms. (**a**) Direct-activated GR binds directly to GREs or ngREs on DNA; (**b**) Tethering—GR acts by bundling with other DNA−binding transcription factors; (**c**) Composite—GR binds directly to DNA and interacts with adjacent DNA-binding transcription factors.

After entering the nucleus, gene expression is regulated by (1) binding to cis-acting elements (glucocorticoid-responsive elements, GREs), (2) “tethering” to other DNA-bound transcription factors, and (3) “composite regulation,” whereby GR starts interacting with other nearby DNA-bound transcription factors after attaching to GREs [25]. The regulatory pathway of GR is shown in Figure 10.

#### 3.2.2. Anti-Inflammatory Role of GCs

**Figure 11:**
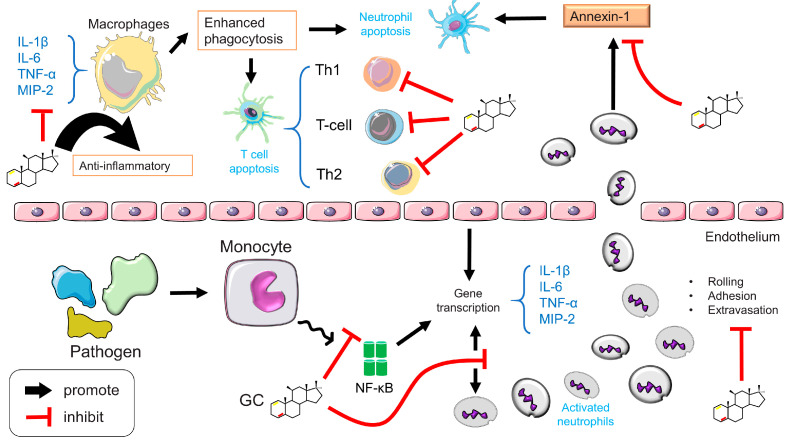
Anti-inflammatory effects of glucocorticoids. During the invasion of pathogens, the immune system will be rapidly activated. In addition to inhibiting the expression of pro-inflammatory factors and immune cells from regulating the inflammatory response, GCs can also inhibit the expression of intercellular adhesion molecule-1 (ICAM-1). It is expressed to prevent extravasation, rolling, and adhesion of neutrophils. In addition, the synthesis of annexin-1 can promote the detachment and apoptosis of neutrophils. GCs inhibit inflammation by inhibiting annexin-1. GCs can also induce T cell death by blocking the production of Th1 and Th2-derived cytokines, thereby converting pro-inflammatory macrophages into an anti-inflammatory effect and increasing their phagocytic function.

The discovery of the anti-inflammatory properties of GCs represents a significant advancement in the treatment of inflammatory illnesses and both natural and synthetic GCs are used extensively as anti-inflammatory drugs (Figure 11) [77,84].

##### Suppression of Pro-Inflammatory Gene Expression by GCs

GCs primarily exert their anti-inflammatory effects by inhibiting the expression of pro-inflammatory chemokines, cell adhesion molecule (CAM), inflammatory enzymes, inflammatory receptors, and cytokine genes. This process helps to restore the equilibrium between pro- and anti-inflammatory activities [85,86].

The current research on the anti-inflammatory effect of GCs is primarily centered on the inhibition of activator protein 1 (AP-1) and nuclear factor-kappa B (NF-κB) transcription. By “tethering” themselves to these genes, GCs suppress the expression of these inflammatory transcription factors and reduce inflammation, a process known as “trans-repression”. GCs accomplish this by binding to the Jun subunit of AP-1 and the p65 component of NF-κB [87,88].

In either its homo- or heterodimeric form, AP-1 serves as one of the primary mediators of the inflammatory response. Its components include Jun (Jun B, Jun D, c-Jun, and v-Jun), the basic leucine-zipper transcription factors, Fos (Fra-1, Fra-2, cFos, and Fos B), MAF (MAFA, MAFF, MAFB, MAFK, NRL, MAFG, and c-MAF), and activating transcription factors (JDP-1, JDP-2, ATF2, ATF3, and B-ATF) [89,90]. The cFos/c-Jun heterodimer is the most prevalent form of AP-1 induced by inflammatory cytokine signaling. Active AP-1 attaches to its response element and regulates pro-inflammatory gene expression [91,92]. The five-member NF-κB family, NF-κB1 (p50/p105), NF-κB2 (p50/p100), RelA (p65), RelB, and c-Rel, is also involved in the initiation and amplification of pro-inflammatory signals. The NF-κB complex and the inhibitory IκB protein family are typically maintained in the cytoplasm in a non-covalent manner. When pro-inflammatory signaling occurs, IκB kinase (IKK) is activated and NF-κB/IκBα is phosphorylated. NF-κB degradation and NF-κB/IκBα phosphorylation cause NF-κB to be released and translocated to the nucleus, where it binds to its response element and controls the production of pro-inflammatory mediators [93].

Once activated, many inflammatory cytokines and chemokines, including TNF-α, IL-1β, IL-6, IL-8, and MCP-1, are released into the blood [94,95,96]. The activation of AP-1 and NF-κB are critical to the pathophysiology of ALI because the inflammatory response extends from alveolar macrophages (AMs) to other cells in the lung [97,98]. Cao et al. [99] performed an electrophoretic mobility shift assay (EMSA) to assess AP-1 and NF-κB expression in the AMs of piglets with ALI. Nitric oxide (NO) and GCs were shown to inhibit AP-1 and NF-κB activation and block inflammation both in vitro and in vivo.

Several additional factors, including T-box expressed in T cells (T-bet), interferon regulatory factor 3 (IRF3), and GATA3, which contribute to inflammatory responses, can modify their transcriptional activity when GR is present. However, further genome-wide studies are required to determine the mechanisms of action [100,101,102,103,104,105].

##### Regulation of Signal Transduction by GCs

GCs can also exert their anti-inflammatory effect by inducing mitogen-activated protein kinase (MAPK) phosphatase 1 (MKP-1), inducing and activating annexin I and repressing cyclooxygenase 2 (Cox-2) to inhibit prostaglandin production [86].

(1) Induction of MAPK-1: MAPKs, including extracellular signal-regulated kinase (Erk 1/2), c-Jun N-terminal protein kinase (JNK), and p38 MAPK, are serine/threonine protein kinases that also mediate signal transduction [106]. The activation of p38 MAPK signaling is involved in regulating inflammation, affecting the severity of AP, in which dominance of NF-κB activation and regulation occurs during SAP progression [107]. In addition to mediating transcriptional changes in gene expression in response to pro-inflammatory stimuli, p38 MAPK positively regulates the stability of multiple pro-inflammatory mRNAs, such as interleukin 6 (IL-6), interleukin 8 (IL-8), Cox-2, tumor necrosis factor-alpha (TNF-α), and vascular endothelial growth factor [108,109,110,111,112,113,114,115,116]. Thus, inhibiting the p38 MAPK signaling may serve as a selective intervention to reduce neutrophil-induced inflammation [117]. GCs inactivate the MAPK protein family members p38, JNK, and Erk 1/2 MAPK by inducing MKP-1 dephosphorylation [86]. MKP-1, one of the three MAPK protein family members, preferentially acts on stress-activated protein kinases and establishes a persistent relationship with p38 both in vivo and in vitro [118,119,120,121]. Lasa et al. [122] found that MKP-1 is the primary factor responsible for mediating DEX-induced suppression of p38 activity and Cox-2 gene expression.

(2) Induction and activation of Annexin I: Annexin I, or “lipocortin-1”, is an anti-inflammatory protein that binds to and inhibits the anti-inflammatory activity of cytosolic phospholipase A2 (cPLA2) [123,124,125,126]. CPLA2 hydrolyzes cellular phospholipids and liberates arachidonic acid and lysophospholipids, which serve as precursor substrates for PAF and eicosanoid production [127]. In dogs with AP, cPLA2 activity is significantly increased in pancreatic tissue, initiating and exacerbating the inflammatory cascade [128,129].

Takahide et al. found that cPLA2 plays an important role in ALI pathogenesis and may be responsible for the generation of the leukotrienes and thromboxane required to mediate ALI [130]. GCs induce the production of Annexin I, which exerts its anti-inflammatory effects by preventing cPLA2 from inhibiting the release of arachidonic acid and its eicosanoids, prostacyclins, prostaglandins, leukotrienes, and thromboxanes [131,132,133].

**Figure 12:**
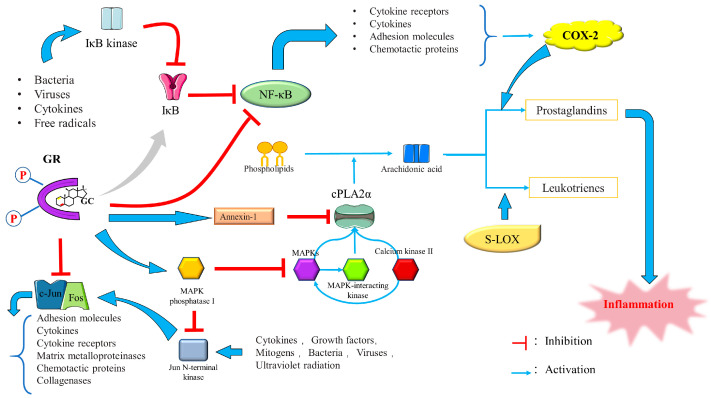
Anti-inflammatory signaling mechanisms of GCs. Blue line: induction; red line: inhibition; Enzyme: cPLA2Aα, COX-2, A-LOX; Protein kinase: IκB kinase, Jun N-terminal kinase, MAPKs, MAPK-interacting kinase, Calcium kinase II; Inflammatory transcription factor: c-Jun, Fos, NF-κB; Protein phosphatase: MAPK phosphatase I; Inhibitory protein: IκB, Annexin I; GC receptors inhibit these inflammatory pathways by blocking NF-kB and inducing the expression of anti-inflammatory proteins such as Annexin I, MAPK phosphatase I, and IκB.

Repression of COX-2 by GCs: COX-2 plays a critical role in the inflammatory response and suppression of this gene can significantly reduce AP severity [134]. COX-2 expression is partially regulated by NF-κB and inhibited by anti-inflammatory cytokines, such as transforming growth factors, interleukin 4 (IL-4), and IL-10 [135,136,137,138]. Hiroyasu et al. [139] found that DEX mediates the dose-dependent suppression of COX-2 activity under the control of GR. However, in the absence of cotransfection, this inhibition can also occur at the posttranscriptional level. Preventing COX-2 production is the third way of inhibiting prostaglandin production by GCs [140]. The anti-inflammatory signaling mechanisms of GCs are shown in Figure 12.

##### Novel Anti-Inflammatory Mechanisms

Recent research has found unique anti-inflammatory modes of action of GCs, contributing to the reinterpretation and development of modern anti-inflammatory medications. We can design innovative anti-inflammatory medications for the treatment of autoimmune and inflammatory disorders using these new targets and methods of control, with lower toxicity and adverse effects.

After the cloning and isolation of the human homologue of murine GILZ (mGILZ), a novel substance, glucocorticoid-induced leucine zipper (GLIZ), was deciphered from the laboratory. This molecule is a protein consisting of 135 amino acids with a molecular mass of about 15 kDa, capable of regulating T cell activation and death and contributing to the anti-inflammatory impact of GCs in leukocytes. Distributed broadly in immune organs such as the bone marrow, thymus, spleen, and lymph nodes, GILZ suppresses inflammatory responses in numerous immune cell lineages and has effects comparable to GCs in lowering disease severity in animal models of inflammation [141,142]. In the case of T cells, epithelial cells, bone marrow cells, and mast cells, GCs have the impact of elevating GILZ expression. Inhibition of LPS-activated macrophages, suppression of NF-kB gene transcription in T cells and macrophages, and suppression of the aforementioned AP-1 and MAPK inflammatory pathways are all mechanisms by which the increased expression of GILZ exerts its anti-inflammatory effects [143]. In the regulation of GILZ in vasculitis, for instance, it was discovered that the expression of the anti-inflammatory mediator GILZ was decreased in inflamed vessels as a result of the mRNA-binding protein ZFP36, which prevented the progression of vasculitis by inhibiting NF-kB activation in endothelial cells [144]. Tristetraprolin (TTP) was first identified as a protein that is transiently activated by mitogen and growth factor stimulation of fibroblasts. This recently discovered protein detects certain transcripts and destroys the mRNAs of a variety of pro-inflammatory cytokines using exonucleases. Its structure is composed of tandem CCCH zinc fingers (TZFs) with well-conserved sequences and spacing. TTP binds to AU sites in mRNA through the TZF structure, hence boosting mRNA transcription and removing the poly(A) tail from mRNA [145]. Several inflammatory factors, including colony-stimulating factor-2 (CSF-2), interleukin-2 (IL-2), and COX-2, are differentially expressed in TTP deficiency. Phosphorylation of TTP by mitogen-activated protein kinase 2 reduces its activity and increases the expression of pro-inflammatory mRNAs [146]. Human keratinocytes, HeLa cells, and lung epithelial cells (BEAS-2B and A549) were stimulated once exogenous GCs were added, leading to a rise in activity and an increase in TTP mRNA expression. This suggests that the activated expression of TTP genes during post-transcriptional regulation may be responsible for the inflammatory suppressive effect of GCs [143].

##### Other Anti-Inflammatory Pathways Regulated by GCs

GCs can also impact inflammation by being immunosuppressive, influencing the activity of other kinases, interacting non-specifically with membrane components, or attaching to the membrane-bound glucocorticoid receptor (mGCR).

GCs can regulate immune responses at either the cellular or the molecular level. (1) Cellular level: GCs inhibit lymphocyte maturation, induce basophilic and eosinophilic apoptosis, and maintain dendritic cells (DCs) in an immature state. GCs can also induce T cell redistribution and apoptosis and mediate T cell transformation from a Th1 to a Th2 phenotype. (2) Molecular level: GCs inhibit MHC II expression by reducing the interaction between immature T cells and DCs, limiting the expression of pro-inflammatory factors such as TNF-α, IL-1β, and IL-6, and increasing the expression of anti-inflammatory factors, such as transforming growth factor-α (TGF-α) and IL-10 [147].

GR also exerts its anti-inflammatory effects by regulating kinases, enzymes that can phosphorylate S, Y, or T residues on proteins and thereby alter their structure, function, and metabolism [148]. Inflammatory signaling involves multiple mechanisms. Some kinases, including MAPK, MSK, Cdks, IKKα, and TBK1, regulate inflammatory cytokine production. Activated GR plays a pivotal role in inhibiting kinase activity [149]. (1) MSK: In the complex regulatory circuit formed by MAPK and GR, following the inhibition of p38, JNK, and Erk 1/2 MAPK phosphorylation, MAPKs also activate MAPK-activated kinases (MKs) such as mitogen- and stress-activated protein kinase (MSK), MK2, MK3, MK5, 90-kDa ribosomal S6 kinase (RSK), and MAPK-interacting kinases (MNK) [150]. MSK proteins are divided into MSK1 and MSK2, which consist of two domains. GCs inhibit MSK1 protein not by inhibiting its phosphorylation but by driving MSK1 protein into the cytoplasm in a manner that prevents MSK1 recruitment at the initiation of inflammatory genes and a CRMI1-dependent activity [151,152]. There are few studies on MK2 and the association between GCs and other MKs, such as MK3, MK5, and MNK, remains unknown. (2) Cyclin-dependent kinases (Cdks): GCs inhibit Cdk2 and Cdk4 which reduces GRE-mediated transcription. Cdk4, Cdk6, and cyclin D3 are also inhibited, and expression of the Cdk inhibitor, p21Cip1, is further induced [153,154,155,156,157,158]. (3) IKKα: IKK is a multifactorial kinase complex that is indispensable in the pro-inflammatory activation of NF-κB. The ubiquitin–proteasome degradation process degrades phosphorylated IKK, after which NF-κB is released into the nucleus, facilitating its binding to gene promoters [159,160]. GCs induce IκBα expression, reversing IκBα depletion and counteracting the effect of IKKα on NF-κB [161,162]. (4) TANK-binding kinase 1 (TBK1): TBK1 is an NF-κB activator related to a TRAF family member. After TLR3 (Toll-like receptor 3) and TLR4 (Toll-like receptor 4), the downstream signaling pathway is engaged and, together with the IκB kinase IKKε, it functions as an intermediary in the activation of the transcription factor IRF3. GCs can mediate the inhibition of TBK1 [163,164,165]. Song et al. [166] found that inhibition of STING/TBK1/IRF3 signaling prevents AP-associated inflammation. (5) GCs also have a regulatory effect on rho-dependent protein kinase 2 (ROCK 2) and every member of the Src family of tyrosine kinases. While ROCK2 activation is associated with lung inflammation, c-Src kinase promotes IκBα phosphorylation and NF-κB translocation [167,168,169].

Increasing evidence suggests that GCs can elicit cellular responses within minutes through a rapid response action that cannot be explained by traditional genomic mechanisms [170,171,172,173,174]. Stahn et al. [175] found that at high concentrations, GCs are directly embedded into the plasma and mitochondrial membranes of immune cells and interfere with transmembrane circulation. This immediately alters the physicochemical features of the membrane structure and the activity of membrane-associated proteins and decreases the calcium and sodium cycling of the cytoplasmic membrane, which contributes to rapid immunosuppression and inflammatory response processes [176,177].

MGCR is a cGCR that is transported to the cell membrane and is also formed by post-translational editing of cGCR variants [178]. The receptor was first discovered in lymphoma cells and in the cell membranes of amphibian neurons. GCs induce mGCR-mediated apoptosis, which inhibits inflammation [179].

#### 3.2.3. Microcirculation Improvement by GCs

Microcirculation dysfunction can aggravate AP and induce intestinal barrier damage and ALI [180]. The mechanisms for pancreatic and systemic microvascular disturbances that occur during AP progression include ischemia–reperfusion injury, oxygen-derived free radical production, increased blood viscosity, microvascular damage, and increased capillary permeability [181].

The pancreatic blood supply determines the susceptibility of pancreatic tissue to ischemia [182]. Ischemia and hypoperfusion are critical early stages of the disease that transform edematous pancreatitis into hemorrhagic and necrotizing pancreatitis [183,184]. Reperfusion injury following ischemia disrupts acinar architecture, activates intracellular proteases, and induces oxygen-free radical production [185]. Free oxygen radicals, including malondialdehyde, conjugated dienes, xanthine oxidase, malondialdehyde, and conjugated dienes in serum and tissues react with phospholipids in the cell membrane and cause cell damage. At the same time, chemokines cause the recruitment and margination of polymorphonuclear leucocytes [186,187]. Leukocyte adhesion and migration and ICAM-1 expression are all regulated by oxygen-free radicals. Research indicates that ICAM-1 is an important mediator of adhesion molecules during AP [188,189,190,191]. Intravascular thrombosis is present in the pancreas and the lungs of AP patients and this is associated with elevated D-dimer, fibrinogen, and platelets in the diseased tissue. Coagulation and increased blood viscosity exacerbate the microcirculatory disturbance caused by AP and have a profound impact on organ circulation that can cause secondary damage [192,193,194]. Changes in microvascular architecture are observed in the first 30 min of disease, and microvascular casting studies have detected endothelial disruption, manifesting as irregular capillary outlines, vessel fragmentation, and poor vascular filling [195,196,197,198]. Similarly, in AP animal models, microscopy has revealed vessel wall media necrosis, irregularities in the endothelial lining, micropinocytic vesicles, and polymorphonuclear leucocyte infiltration [199,200,201]. Endothelial cell destruction activates neutrophils and pancreatic enzymes, such as phospholipase A2, elastase, and trypsin, increase pancreatic and systemic capillary permeability and alter hemodynamics, leading to the migration of macromolecular proteases into pancreatic tissue and further aggravating cell damage [183,202,203].

GCs enhance the survival rate of rats with AP, increase arterial blood flow in pancreatic tissue, boost cardiac output, improve local blood flow, and restore hemodynamics in the microcirculation [204,205,206]. Laviolle et al. [207] found that GCs play a non-genomic vascular role, inducing vasodilation and contraction that improves hemodynamics and vascular reactivity. After pretreatment with DEX, the suppression of ICAM-1 production by endothelial cells or neutrophils, as well as a reduction in rolling and adherence of leukocytes to postcapillary venules, was seen in experimental models of sepsis [208,209,210,211,212]. Non-genomic processes are thought to play a role in this rapid response. GCs modify the conformational structure of CAM within minutes, changing the surface distribution and affinity status and inhibiting cell-to-cell aggregation [132,213]. GCs also help to maintain the stability of cellular, extracellular basement, and lysosomal membranes, preventing damage after lysosomal release and maintaining vascular structure [214,215,216]. In the pulmonary vasculature, DEX also reduces vascular permeability, improves oxygenation, enhances ventilatory adaptation, and alleviates hypoxia-induced endothelial dysfunction [217,218,219,220]. Murata et al. [219] found that DEX prevents pulmonary vasoconstriction caused by chronic hypoxia by increasing endothelial nitric oxide (eNOS) mRNA expression or enhancing NO efficacy by directly activating eNOS in endothelial cells.

#### 3.2.4. The Protective Effect of GCs on Lung Tissue

Vital organ dysfunction and failure caused by systemic inflammation is a reaction of the body to infectious or non-infectious factors and it is the main reason for increased mortality in ALI/ARDS patients. To reduce the occurrence of ALI/ARDS, restore body temperature, and improve patient survival, it is essential to lower systemic inflammation [221]. In its early stages, ALI/ARDS manifests as acute/diffuse damage to the lung parenchyma (endothelial/epithelial lining of the terminal respiratory unit) and severe exacerbation of the host defense response (HDR). Its progression depends on disruption of the basement membrane, alterations in vascular permeability, exudation of fluid from the alveoli, and the degree of the initial pulmonary HDR [222,223,224,225,226].

The HDR is a concept proposed by Meduri in 1996. It is a protective response that can dilute, destroy, or contain injurious agents and subsequently repair tissue, through the regeneration of native parenchymal cells or the use of fibroblastic tissue to cover deficiencies or replace damaged tissue. There are five types of HDRs during ALI/ARDS, inflammation, coagulation (including intravascular coagulation and extravascular fibrin deposition), immunomodulation, tissue repair, and the activation of the “hypothalamic–pituitary–adrenal (HPA) axis”, which is responsible for GC production [227]. Unregulated production of HDR mediators causes damage to lung tissue structure, the persistence of inflammation, coagulation inside and outside of blood vessels, mesenchymal cell proliferation, and damage to extracellular matrix (EMC) deposition that eventually leads to pulmonary fibrosis [228] and irreparable damage to lung anatomical structure and function, which manifests as fever, sepsis, SIRS, and MODS. Prolonged MV eventually results in the death of lung tissue [229,230,231]. In autopsies of ALI/ARDS patients, 55% had pulmonary fibrosis, and 69% had pneumonia. About 36–90% of ALI/ARDS deaths are related to sepsis and severe nosocomial infections such as ventilator-associated pneumonia (VAP), which amplify SIRS and accelerate the progression of MODS [232,233,234,235,236].

There is no known way to halt the progression of ALI/ARDS. However, by preventing inflammation, inhibiting fibroproliferation, promoting epithelial/endothelial cell repair, and reducing EMC deposition, excessive and persistent HDR suppression can theoretically prevent further damage to lung tissue [227,237]. Studies have demonstrated that the HPA axis is critical to the HDR in ALI/ARDS patients. GCs inhibit the HDR cascade reaction from almost all angles to protect the host from over-regulation, and exogenous GCs drugs enhance this effect [238,239,240,241]. Meduri et al. [242] supported a correlation between the HDR and ARDS progression by treating ARDS patients with GCs and showing the association between reduced inflammatory cytokine production and improved lung function in plasma and bronchoalveolar lavage (BAL). Several studies [243,244,245] have found that GCs can reduce pulmonary edema and lung collagen exudation. Timely GC treatment can also prevent the progression of fibrosis and, when the wound enters the stage of rapid healing, GCs can accelerate collagen biosynthesis and degradation, promoting tissue repair [227].

#### 3.2.5. Immunosuppressive Effects

The development of autoimmune illnesses is linked to HPA axis abnormalities and variations in pathological states, and GC administration may decrease immune activity by interacting with almost all kinds of immune cells, including those in the periphery (e.g., macrophages, dendritic cells, neutrophils, and T cells). First, GCs have the function of regulating the phenotype and activity of monocyte-macrophages and preventing the death of macrophages through an anti-apoptotic effect thus promoting the continuation of their anti-inflammatory effects. Related intrinsic mechanisms include the mediation of MAPK and extracellular signal-regulated kinase (ERK), which promotes the expression of macrophage anti-apoptotic genes and inhibits caspase activity [246]. GCs also reduce the oxidative burst and adherence of macrophages, which improves their phagocytic capacity [247]. Second, GCs inhibit the capacity of dendritic cells (DCs) to excite T cells by inhibiting the expression of cytokines and co-stimulatory molecules and promoting the creation of GILZ. In order to inhibit their immunological activity, GCs control the survival, maturation, and migration of DCs toward lymph nodes through this mechanism [248]. Third, neutrophil extravasation and rolling, adhesion, transmigration, and activation at the site of infection are crucial processes in the inflammatory response, and GCs, as a key neutrophil regulator, participates in and controls all of these phases. GCs modulate neutrophil rolling and adhesion by inhibiting endothelial counterparts and leukocyte integrins, and by reducing the production of ICAM-1, it reduces neutrophil transmigration to the site of infection [132,208]. Fourth, in addition to triggering matrix metalloproteinases, chemokine receptors, and adhesion molecules, GCs decrease the number of T cells in circulating blood by stimulating the migration of T cells back to secondary lymphoid tissues or bone marrow and by enhancing the expression of adhesion molecules. GCs are also engaged in T-cell apoptosis via the dimerization of GR and the stimulation of Bim and Puma expression [249,250]. Helper T cell (Th cells) subpopulations, such as Th1, Th2, Th17, and regulatory T cells (Treg cells) are differentiated from naïve Th cells and perform distinct functions in response to GCs, which may be linked to differences in the expression of GR isoforms [251].

#### 3.2.6. Endothelial Cell Protection

About 2.5 × 10^12^ endothelial cells may be found in a human body, with most of their morphological features belonging to flattened epithelial cells. These cells are mostly found in the vasculature and are intimately coupled [252,253]. Endothelial cells regulate the immune system, regulate blood flow via the vessels, and maintain homeostasis [252,254]. The reaction of endothelial cells is essential in the process of inflammation. Using endothelial cells as targets, GCs are crucial for the physiological control of endothelial cells and the maintenance of the endothelium barrier [255]. Endothelial cells, which normally sit dormant at the blood–tissue interface, become active at the start of an inflammatory reaction. Endothelial cells produce selectins (such as E- and P-selectin) to recruit leukocytes in response to the activation of inflammatory cytokines, and in the presence of vascular cell adhesion molecule-1 (VCAM-1) and intercellular adhesion molecule-1 (ICAM-1), leukocytes can cross the endothelial barrier and extravasate to the site of the inflammatory response [256,257].

GCs’ protective effects on endothelial cells manifest themselves in a number of ways. To begin, GCs inhibit pro-inflammatory signaling pathways, such as the binding of GATA and AP-1 to remaining endothelial cells and the nuclear translocation of NF-B, and it promotes protective proteins that keep the endothelium barrier stable during inflammation. As a result of GC stimulation, endothelial cells release AnxA1, which helps keep the blood–brain barrier (BBB) stable by inhibiting the synthesis of pro-inflammatory mediators through phospholipase A2 [258,259,260]. Second, GCs keep the endothelial barrier strong by lowering the number of leukocytes that can cross it. It does this by decreasing the levels of soluble forms of intercellular adhesion molecule (ICAM)-1, VCAM-1, and E-selectin (sICAM-1, sVCAM-1, and sE-selectin), as well as by inhibiting the production of chemokines and pro-inflammatory cytokines by endothelial cells [261,262]. Third, GCs increase the expression of junctional proteins (including claudin-5, occluding, and VE-cadherin) and eNOS, both of which are important mediators of vascular integrity. Concurrently, matrix metalloproteinase-9 (MMP-9) is inhibited by GCs. This enzyme destroys functional proteins. The activation of this pathway is primarily mediated by the upregulation of TIMP-3 and TIMP-1 [263,264,265].

The earliest cellular event in the pathology of ALI/ARDS indirectly caused by SAP is lung microvascular endothelial cell injury brought on by extrapulmonary stimuli. This injury results in endothelial cell dysfunction, increased vascular permeability, and the release of significant amounts of inflammatory mediators, which results in interstitial edema. In this process, endothelial cell dysfunction and loss of barrier integrity are exacerbated by abnormal proliferation, necrosis, or apoptosis of pulmonary microvascular endothelial cells, as well as relaxation of intercellular junctions. ALI/ARDS development is sped up concurrently by inflammatory factor overexpression and inflammatory cell infiltration. Consequently, in the above description, GCs are essential for maintaining endothelial cell barrier protection, inhibiting inflammatory cell infiltration, and consolidating endothelial cell-to-endothelial cell connections, all of which are crucial prevention strategies for SAP-ALI [266,267,268]. In an in vitro study, Thomas Dschietzig et al. [269] discovered that dexamethasone reduces TNF-α- and IL-1β-induced pulmonary endothelin (ET-1) release by inhibiting NF-κB transcriptional activity, and thus protects the pulmonary vasculature and tissue endothelial cells in ARDS.

## 4. Clinical Use of GC in SAP-ALI/ARDS Treatment

### 4.1. Clinical Treatment for SAP-ALI/ARDS

Organ failure due to early SAP (24–72 h) is primarily aseptic systemic inflammation driven by the release of pro-inflammatory mediators and increased mucosal permeability due to intestinal bacterial translocation, with coagulation disorders, capillary infiltration with edema, myocardial depression, and hemodynamic disturbances determining this process [270]. Pancreatic necrosis and peripancreatic septicemia are the leading causes of late-stage organ failure (>2 weeks) [271]. So, it is crucial to have reliable methods for gauging severity and predicting risks before proceeding with therapeutic care. Although there is room for improvement in the accuracy of all existing prediction systems, monitoring key biomarkers, imaging, hemodynamics, and intra-abdominal pressure may serve as early indications for mortality prediction and as an essential duty throughout the patient treatment process [272,273].

Large capacity fluid recovery of intravenous drug delivery within 24 h (5000 mL) is an essential treatment for early SAP, and the “balance” liquid crystal (Ringer’s lactate) has replaced physiological saline as the preferred recovery liquid. In addition to enhancing pancreas microcirculation, Ringer’s lactate can also reduce the risk of acidosis and pancreatic enzyme activity [274]. Due to the failure of prophylactic antibiotics to influence pancreatic necrosis, enhance the length of stay, or reduce death, they are no longer used regularly to treat SAP-ALI/ARDS. Antibiotics are the therapy of choice only when infections such as lung infections or sepsis arise; thus, empirical evaluation of early illness is the primary criterion for administering preventive antibiotics [275,276]. Hyperlipidemic SAP is often treated with fibrates, statins, and even plasma exchange, whereas biliary SAP is typically treated using endoscopic retrograde cholangiopancreatography (ERCP) to release pressure in the pancreatic duct. Peritoneal lavage, early enteral feeding, hemofiltration, and the use of GCs are other options for therapy [277]. If SAP produces distal lung tissue damage that leads to ALI/ARDS, standard therapy should include mechanical protective breathing, prone position, extracorporeal membrane oxygenation, pulmonary vasodilators, and GC neuromuscular blocking drugs. With a utilization rate of 41.5%, GCs are the most commonly utilized adjuvant therapy among these options [278].

### 4.2. The Application Status of GCs

The use of GCs in AP treatment is controversial. In 1952, the first study on GCs’ efficacy among patients with acute hemorrhagic pancreatitis was published, and since then, several studies have assessed the role of GCs in the treatment of AP. However, little progress has been made in clinical research [279,280]. There are no current guidelines recommending GC treatment for AP patients, although GCs are commonly used to prevent ALI/ARDS, maintain the stability of blood function, and reduce persistent organ damage. Even while GCs are often considered for patients with more severe AP, they are not always utilized to treat the condition [281]. A meta-analysis of corticosteroid treatment for SAP assessed 320 patients from six randomized, controlled trials that occurred in China from 2002 to 2010. Corticosteroids were found to prolong survival and reduce hospital stays among SAP patients and also to reduce surgical intervention, pain, and mortality [282]. Wang et al. [281] conducted a propensity score matching analysis of 818 AP patients hospitalized during 2014–2019 and found that GC administration reduced the mortality, incidence of multiple organ failure, and hospital costs of AP patients, and did not increase the risk of gastrointestinal hemorrhage and infection.

In 2017, the European Society of Intensive Care Medicine (ESICM) and the Corticosteroid Guideline Task Force of the SCCM issued guidelines for GC treatment of critically ill patients. In the absence of other complications, GCs significantly reduced MV (RR = 0.45; 95% CI = 0.2–0.79), shortened hospital stay (risk difference = −2.96, 95% CI = −5.18–−0.75), and prevented ARDS (RR = 0.24;9 5%CI = 0.10–0.56) [283]. Overall, GCs are more effective at reducing symptoms than treating the underlying disease. Thus, an appropriate course of treatment should be determined prior to GC use to ensure that the disease is treated and external symptoms are controlled [10]. Common clinical uses of GCs include replacement GCs, short-term, long-term, pulse, repository, topical, intralesional, and alternate-day therapies [10,283]. The effectiveness and side effects of drugs like GCs are heavily influenced by the treatment duration.

A review of findings from multiple studies found that, after accounting for the duration of the disease (i.e., SIRS and critical illness-related corticosteroid insufficiency, CIRCI), the length of HPA axis restoration following GC cessation, and the related risks of GC treatment, long-term GCs (≥7 days) were considered the most favorable treatment for ALI/ARDS [242,284,285,286,287,288]. GCs inhibit coagulation, inflammation, and fibroproliferation at the tissue level, reduces the alveolar-capillary membrane (ACM) permeability index, and improves organ dysfunction scores in other organs. Meduri et al. [289] found that prolonged MP treatment positively impacted the histological and physiological manifestations of ALI/ARDS and improved GC resistance caused by inflammation at the cellular level. Moreover, the expression of pulmonary inflammatory markers was significantly higher in response to GCs than to other interventions.

### 4.3. Administration of GCs

Based on the pharmacological mechanism of GCs, it is more important to manage the mode of GC administration for clinical patients. The advantages and disadvantages of different modes of administration, including dosage and delivery mode, are discussed. Supplementary Table S1 provides a summary of the different modes.

According to GC medication guidelines published by Meduri et al. in 2020 [4], GCs use for >7 days is defined as long-term administration. A daily dose of 1500 mg HC (equivalent to 300 mg MP, 56.3 mg DEX) is considered a massive dose, 500–1500 mg HC (equivalent to 100–300 mg MP, 18.8–56.3 mg DEX) is considered a medium dose, and a daily dose of GCs below the medium dose is considered a low dose. Of 19 clinical studies, 13 confirmed that GCs benefit SAP-ALI/ARDS patients, and most study results (9 of 13) suggested that intravenous injection and a long-term GC administration time (≥7 days) was the best treatment option. At the same time, low-dose short-acting HC (200 mg/d), medium-dose intermediate-acting MP (100–300 mg), and low-to-medium-dose long-acting DEX (10–20 mg/d) were found to provide the most benefit to patients.

### 4.4. Limitation of GCs

As mentioned above, SAP-ALI/ARDS treatment and prevention are greatly improved by the use of GCs in a sensible and consistent manner. However, excessive or prolonged administration can cause unpredictable damage to the body.

#### 4.4.1. AP Induction by GCs

It is important to note that, while GCs are an effective treatment for SAP, this drug can also induce AP in individuals who have never suffered from this condition. The incidence of such drug-induced AP is only 0.1–2% [290]; however, the mechanism remains unclear and the diagnosis is difficult to determine. High-risk factors for AP, including cholelithiasis, the use of other drugs, binge eating, and alcoholism, are often confounded. In addition, diseases with indications for GC use are also high-risk factors for AP. For example, 8% of individuals with systemic lupus erythematosus (SLE) will develop AP regardless of whether GCs are used [291]. In a nationwide, Swedish registry-based study involving 6161 AP and 61,637 control patients, oral GC users had a higher chance of acquiring AP, and this was the most pronounced 4–14 days after treatment [292]. Yoshizawa Y et al. [293] found that high-dose corticosteroid pulse therapy caused an increase in serum amylase and induced corticosteroid-induced pancreatitis (CIP).

#### 4.4.2. Infection Caused by GCs

Several factors are responsible for opportunistic infections caused by GC treatment, including the route of administration and drug dose, efficacy, and duration. Long-term and high dosage systemic GC therapy has been shown to enhance infection risk in several trials. MacGregor et al. [294,295] confirmed that GC alternate-day therapy in patients with schizophrenia significantly reduced the risk of infection. This side effect is mainly related to immune dysregulation. GCs can affect white blood cell levels and lead to reversible monocytopenia and lymphopenia. In addition, GCs can disrupt immune cell function by suppressing lymphocyte production and migration, T lymphocyte activation and proliferation, natural killer cell toxicity, and macrophage phagocytosis, as well as by limiting oxidative killing and phagocytosis [296].

#### 4.4.3. Hemorrhage Caused by GCs

The correlation between GCs and gastrointestinal bleeding remains controversial. Conn et al. [297] found no evidence that GC treatment increased the risk of bleeding in the digestive tract, while Messer et al. [298] argued that steroid use was associated with digestive tract bleeding. The occurrence of digestive tract bleeding and peptic ulcers are closely related and dose dependent. Luo et al. [299] observed that DEX slowed ulcer healing by suppressing vascular endothelial growth factor and prostaglandin E2 production.

A meta-analysis of 159 trials found that corticosteroid treatment was associated with a 40% increase in the OR value for gastrointestinal bleeding, which is linked to poor stomach tissue regeneration and delayed wound healing [300].

#### 4.4.4. Other Effects of GCs

It is important to highlight that GC substance abuse is a primary cause of non-traumatic osteonecrosis. According to a study of 3000 patients conducted in 1998, more than a third of non-traumatic osteonecrosis patients were treated with corticosteroids. In recent years, corticosteroid-associated osteonecrosis has been shown to involve oxidative stress, endothelial cell damage, coagulation, apoptosis, and autophagy, in addition to inflammatory signaling pathways, such as PERK, Parkin, and PDK1/AKT/mTOR [301]. Koo et al. found that the GC dose required to cause corticosteroid-associated osteonecrosis was 1800–15,505 mg of prednisolone (or its corresponding GC dose), with an average intake of 5928 mg [302]. The incidence increased with higher treatment doses and duration. Typically, osteonecrosis is only triggered by >3 months of steroid use. Irregular use of GCs can also cause Cushing’s syndrome and diabetes. After several weeks of GC treatment, symptoms like alopecia, poor wound healing, and muscle weakness appear in experimental animals [10,303,304]. Yubero et al. [305] confirmed that, while DEX can reduce serum amylase levels in SAP patients and inhibit the upregulation of lung tissue P-selectin, CINC, MCP-1, and ICAM-1, this drug fails to have a beneficial effect on leukocyte infiltration and lung histology. Recent research discovered that GCs may cause disorders like myocardial infarction and acute renal failure through a sensitizing mechanism that promotes iron death. Iron death is a kind of programmed cell death mediated by iron and predominantly induced by glutathione (GSH) deficiency. GSH metabolism regulating protein dipeptidase-1 (DPEP1), an enzyme that inhibits GSH levels, has been involved in similar investigations; dexamethasone has been found to improve susceptibility to iron-induced cell death by way of GR-mediated overexpression of DPEP1 [306].

## 5. Summary

The application and management of GC drugs are important topics in the medical field. Many in vivo and in vitro experiments have confirmed the powerful anti-inflammatory effects of GCs at the genetic, protein, and molecular levels. GCs have prevented further progression of AP to sepsis and ALI/ARDS by altering microcirculatory disorders, improving intestinal barrier damage, inhibiting cytokine storms, modulating immunity, and promoting tissue repair. The studies discussed in this review suggest that (1) GCs are a “double-edged sword” and their advantages and adverse effects need to be weighed before using in clinical practice; (2) the number and function of circulating immune cells in SAP patients require regular monitoring and GC therapy should be administered with clear indications; (3) low-to-moderate GC dosages given intravenously are most effective for individuals with sepsis and early SAP-ALI but are not necessarily ideal for all conditions; and (4) constant evaluation of the patient’s treatment response and vigilant monitoring of blood glucose and electrolyte levels as well as central system abnormalities should follow GC therapy. Additional clinical and experimental evidence-based studies are still required to guide the conditions and timing of GC application and prevent the increased risk of death from careless use.

Finally, it is critical to continue researching the efficacy of GCs in treating SAP-associated lung damage and sepsis since no other medications are available to address these conditions. This is especially important given its associated hazards. In addition, mechanical insights point to the possibility of a breakthrough in the development of new drugs that target the modulation of the pro-/anti-inflammatory balance as an alternative to GCs.

## Data Availability

Not applicable.

## Supplementary Materials

The following supporting information can be downloaded at: https://www.mdpi.com/article/10.3390/ijms241512138/s1. Refs. [307,308,309,310,311,312,313,314,315,316] are cited in Supplementary Materials.

## Abbreviations

ALI/ARDS: acute lung injury/acute respiratory distress syndrome; SIRS: systemic inflammatory response syndrome; SAP: severe acute pancreatitis; GC: glucocorticoid; CRH: corticotrophin-releasing hormone; AVP: arginine vasopressin; ACTH: adrenocorticotrophic hormone; HPA: Hypothalamic-Pituitary-Adrenal; GABA: γ-aminobutyric acid; MC: aldosterone; DHEA: dehydro-epi-androsterone; GR: glucocorticoid receptor; AR: androgen receptor; ER: estrogen receptor; PR: progesterone receptor; MR: mineralocorticoid receptor; NR: nuclear receptor; CBG: corticosteroid-binding globulin; GREs: glucocorticoid response elements; 11β-HSD: 11β-hydroxysteroid dehydrogenase; MC: mineralocorticoids; MP: Methylprednisolone; DEX: Dexamethasone; CI: confidence interval; MODS: multi-organ dysfunction syndrome; TNF-α: tumor necrosis factor-α; IL-1β: interleukin-1β; IL-6: interleukin-6; IL-10: interleukin-10; ICAM-1: Intercellular adhesion molecule-1; PAF: platelet-activating factor; H2S: hydrogen sulfide; CARS: compensatory anti-inflammatory response syndrome; MARS: mixed anti-inflammatory response syndrome; NTD: N-terminal transactivation domain; DBD: DNA-binding domain; LBD: ligand-binding domain; PTMs: post-translational modifications; AF1: activation function-1; AF2: activation function-2; AP-1: activator protein 1; NF-κB: nuclear factor-kappa B; IKK: IκB kinase; EMSA: electrophoretic mobility shift assay; AM: alveolar macrophages; NO: nitric oxide; T-bet: T-box expressed T cells; IRF3: interferon regulatory factor 3; MAPKs: mitogen-activated protein kinases; JNK: c-Jun N-terminal protein kinase; Erk 1/2: extracellular signal-regulated kinase; IL-8: interleukin 8; Cox-2: cyclooxygenase 2; MKP-1: MAPK phosphatase 1; cPLA2: cytosolic phospholipase A2; IL-4: interleukin 4; GILZ: Glucocorticoid induced leucine zipper; mGILZ: murine GILZ; TTP: Tristetraprolin; TZF: tandem CCCH zinc fingers; CSF-2: colony-stimulating factor-2; IL-2: interleukin-2; Tregs cells: regulatory T cells; Th cells: Helper T cell; VCAM-1: vascular cell adhesion molecule-1; BBB: blood-brain barrier; MMP-9: matrix metalloproteinase-9; GSH: glutathione; DPEP1: GSH metabolism regulating protein dipeptidase-1; mGCR: membrane-bound glucocorticoid receptor; DCs: dendritic cells; TGF-α: transforming growth factor-α; MKs: MAPK-activated kinase; MSK: mitogen- and stress-activated protein kinase; MNK: MAPK-interacting kinases; Cdks: Cyclin-dependent kinases; TBK1: TANK-binding kinase 1; TLR3: Toll-like receptor 3; TLR4: Toll-like receptor 4; ROCK 2: Rho-dependent protein kinase 2; CAM: cell adhesion molecule; eNOS: endothelial nitric oxide; HDR: host defense response; EMC: extracellular matrix; VAP: ventilator-associated pneumonia; BAL: bronchoalveolar lavage; ESICM: European Society of Intensive Care Medicine; SCCM: Society of Critical Care Medicine; CIRCI: critical illness-related corticosteroid insufficiency; ACM: alveolar-capillary membrane; HC: hydrocortisone; MV: Mechanical Ventilation; DCQD: modified Dachengqi Decoction; SLE: systemic lupus erythematosus; CIP: corticosteroid-induced pancreatitis.
