# Supplementary material for: Glucocorticoid Treatment in Acute Respiratory Distress Syndrome: An Overview on Mechanistic Insights and Clinical Benefit
Source: Int J Mol Sci. 2023 Jul 28;24(15):12138. doi: 10.3390/ijms241512138 (PMC10418884; doi:10.3390/ijms241512138)
Supplement: Supplementary file 1 [file ijms-24-12138-s001.zip › ijms-2489883-supplementary.pdf]

Table S1：Summary of clinical application of GC

Drug	Source of literature	Type of literature	Time of administration	Number of patients	Duration of administration（d）	Mode of administration	Dosage	Result
Short-acting								
HC	Annane D et al[1] 《Crit Care Med》——2006	Multiple-center, placebo-controlled, randomized, double-blind study	Early ARDS	300	7	Iv	50mg/6h & 50 g of 9-α-fludrocortisone/d	Enhanced day 28 survival and ICU and hospital discharge, as well as reduced the length of MV.
HC	Nirmeen A et al[2] 《Pharmacology & Pharmacy》——2011	Randomized，double-blind，interventional study	Before ARDS	80	7	Iv	200 mg/d	Significantly reduced length of mechanical ventilation (MV) and improved pneumonia recovery while preventing the development of sepsis-related problems， but had no effect on death.
HC	Confalonieri M et al[3] 《Am J Respir Crit Care Med》——2005	Randomized, double-blind, placebo-controlled trial	Before ARDS	46	7	Iv	200 mg/d<br>Rate：10 mg/h.	A large increase in PaO_2:FIO_2 and the score on the chest radiograph, in addition to an overall decrease in the levels of C-reactive protein, the MODS score, and the severity of delayed septic shock. The use of HC

								therapy was related with a substantial decrease in both the length of time spent in the hospital and the risk of death.
HC	Marik P et al[4] 《Chest》——1993	Randomized placebo-controlled study.	Before ARDS	30	-	Iv	10 mg/kg	Serum TNF- levels and the clinical course of individuals with severe community-acquired pneumonia were unaffected by the administration of HC prior to antibiotic therapy.
HC	Tongyoo S et al[5] 《Critical Care》——2016	Double-blind, single-center, randomized, placebo-controlled trial.	Early ARDS	197	7	Iv	50mg/6h	HC therapy was related with a considerable improvement in pulmonary physiology, but not with a huge rise in survival in sepsis-associated ARDS patients.
Medium-acting								
MP	Bernard GR et al[6] 《The New England journal of medicine》——1987	Prospective，randomized，double-blind，placebo-controlled trial.	Early ARDS	99	-	Ivgtt	30mg/kg	High-dose MP had little bearing on the results in patients with established ARDS caused by infection, aspiration, or a combination of causes.
MP	Keel JB et al[7] 《Respiration; international review of thoracic diseases》——1998	Retrospective study	ARDS after 15 days of MV	31	8	-	100-250 mg（1-3d）<br>80-180 mg（4-8d）	Corticosteroid therapy is beneficial in patients with established fibroproliferative ARDS.

MP	Steinberg KP et al[8]<br><br>《The New England journal of medicine》——2006	multicenter, randomized controlled trial	Late ARDS	180	7-25	Iv	2 mg/kg （single dose）<br>0.5 mg/kg/6h（1-14d）<br>0.5 mg/kg/12h （15-21d）	Despite improved in cardiopulmonary physiology, our data do not support the regular use of MP in the treatment of chronic ARDS. MP treatment may also raise the chance of mortality if it's started more than two weeks after symptoms begin.
MP	Lee HS et al[9]<br><br>《The Annals of thoracic surgery》——2005	Prospective clinical study	Early ARDS	12	-	Iv/Po	2 mg/kg/6h	Initial low-dose steroid treatment inhibits fibroproliferation, an early reaction to lung damage, without affecting operational wound healing.
MP	Meduri GU et al[10]<br><br>《Chest》——1995	Prospective clinical study	Late ARDS	9	42	Iv	200 mg （initial bolus）<br>2-3 mg/kg/d	Long-term corticosteroid rescue therapy has been linked to decreased plasma and BAL IC levels and improved indicators of endothelial permeability and LIS in patients with late-onset ARDS who had a poor chance of survival.
MP	Varpula T et al[11]<br><br>《Intensive care medicine》——2000	Retrospective study	The late phase of ALI	31	-	Iv	80 mg in the morning and 40 mg in the evening	Ten days after the initiation of mechanical ventilation, steroid treatment increases exchange of gases and is related with a reduction in multisystem dysfunction in patients with primary ALI.

MP	Weigelt JA et al[12] 《Archives of surgery》——1985	Randomized double-blind trial	Before ARDS	81	2	-	30 mg/kg/6h	There is no proof to substantiate the use of MP for ARDS in these studies. Steroids did not enhance pulmonary function and were linked to an increased risk of infection in the study population.
MP	Meduri GU et al[13] 《Chest》——2007	Randomized, double-blind, placebo-controlled trial	Early severe ARDS	91	28	Ivgtt	1 mg/kg/d	Down-regulation of systemic inflammation caused by MP was linked to substantial improvements in pulmonary and extrapulmonary organ failure, as well as shorter MV and ICU stays.
MP	Meduri GU et al[14] 《JAMA》——1998	Randomized, double-blind, placebo-controlled trial.	Unresolving ARDS	24	32	Iv/ Po	2 mg/kg/d（1-14d）; 1 mg/kg/d（15-21d）; 0.5 mg/kg/d（22-28d）; 0.25 mg/kg/d（29-30d）; 0.125mg/kg/d（31-32d）	Long-term use of MP in patients with unresolving ARDS was related with a decrease in MODS scoring and a decrease in mortality in this research.
MP	Dumot JA et al[15] 《The American journal of	Randomized, Multicenter, Placebo-	ERCP-Induced Pancreatitis	255	15-30min	Iv	125 mg	It has been shown that administering MP intravenously does not help prevent pancreatitis caused by ERCP.

	gastroenterology》——1998	Controlled Clinical Trial						
Prednisone	Budzyńska A et al[16] 《Endoscopy》——2001	Prospective，randomized，placebo-controlled trial	ERCP-Induced Pancreatitis	300	15h and 3h prior to ERCP	Po	40 mg & allopurinol	There was no evidence that either prednisone or allopurinol had a positive effect on the risk of developing post-ERCP pancreatitis or its severity.
Long-acting								
DEX	Villar J et al[17] 《 The Lancet. Respiratory medicine》——2020	Multicentre, randomized controlled trial	Early ARDS	277	10	Iv	20 mg/d（1-5d）；10 mg/d（6-10d）	When given early on, DEX has the potential to shorten the length of mechanical ventilation (MV) and total mortality in patients who have already been diagnosed with moderate to severe ARDS.
DEX	Wang ZF et al[18] 《World journal of gastroenterology》——2004	-	SAP	32	3-5	-	0.5-1 mg/kg/d & dextran 40 500-1000 ml/d for 7 d.	DEX and dextran 40 decrease the pathological process of SAP by inhibiting proinflammatory cytokines and treating microcirculatory abnormalities, respectively.
DEX	Wan MH et al[19] 《Chinese journal of integrative medicine》——2011	Prospective case-control study	SAP	81	3	Iv	1 mg/kg/8h & modified Dachengqi Decoction（DCQD）	When paired with DCQD, DEX may be able to reduce the likelihood of ARDS developing in SAP patients who are experiencing SIRS and may also shorten the time of their hospitalisation.

References

[1] Annane D, Sébille V, Bellissant E, et al. Effect of low doses of corticosteroids in septic shock patients with or without early acute respiratory distress syndrome[J]. Crit Care Med, 2006,34(1):22-30. doi:10.1097/01.ccm.0000194723.78632.62.

[2] Sabry NA, Omar EE. Corticosteroids and ICU Course of Community Acquired Pneumonia in Egyptian Settings[J]. Pharmacology & Pharmacy, 2011,2(2):73-81.

[3] Confalonieri M, Urbino R, Potena A, et al. Hydrocortisone infusion for severe community-acquired pneumonia: a preliminary randomized study[J]. Am J Respir Crit Care Med, 2005,171(3):242-248. doi:10.1164/rccm.200406-808OC.

[4] Marik P, Kraus P, Sribante J, et al. Hydrocortisone and tumor necrosis factor in severe community-acquired pneumonia. A randomized controlled study[J]. Chest, 1993,104(2):389-392. doi:10.1378/chest.104.2.389.

[5] Tongyoo S, Permpikul C, Mongkolpun W, et al. Hydrocortisone treatment in early sepsis-associated acute respiratory distress syndrome: results of a randomized controlled trial[J]. Crit Care, 2016,20(1):329. doi:10.1186/s13054-016-1511-2.

[6] Bernard GR, Luce JM, Sprung CL, et al. High-dose corticosteroids in patients with the adult respiratory distress syndrome[J]. N Engl J Med, 1987,317(25):1565-1570. doi:10.1056/NEJM198712173172504.

[7] Keel JB, Hauser M, Stocker R, et al. Established acute respiratory distress syndrome: benefit of corticosteroid rescue therapy[J]. Respiration, 1998,65(4):258-264. doi:10.1159/000029273.

[8] Steinberg KP, Hudson LD, Goodman RB, et al. Efficacy and safety of corticosteroids for persistent acute respiratory distress syndrome[J]. N Engl J Med, 2006,354(16):1671-1684. doi:10.1056/NEJMoa051693.

[9] Lee HS, Lee JM, Kim MS, et al. Low-dose steroid therapy at an early phase of postoperative acute respiratory distress syndrome[J]. Ann Thorac Surg, 2005,79(2):405-410. doi:10.1016/j.athoracsur.2004.07.079.

[10] Meduri GU, Headley S, Tolley E, et al. Plasma and BAL cytokine response to corticosteroid rescue treatment in late ARDS[J]. Chest, 1995,108(5):1315-1325. doi:10.1378/chest.108.5.1315.

[11] Varpula T, Pettilä V, Rintala E, et al. Late steroid therapy in primary acute lung injury[J]. Intensive Care Med, 2000,26(5):526-531. doi:10.1007/s001340051199.

[12] Weigelt JA, Norcross JF, Borman KR, et al. Early steroid therapy for respiratory failure[J]. Arch Surg, 1985,120(5):536-540. doi:10.1001/archsurg.1985.01390290018003.

[13] Meduri GU, Golden E, Freire AX, et al. Methylprednisolone infusion in early severe ARDS: results of a randomized controlled trial[J]. Chest, 2007,131(4):954-963. doi:10.1378/chest.06-2100.

[14] Meduri GU, Headley AS, Golden E, et al. Effect of prolonged

methylprednisolone therapy in unresolving acute respiratory distress syndrome: a randomized controlled trial[J]. JAMA, 1998,280(2):159-165. doi:10.1001/jama.280.2.159.
[15] Dumot JA, Conwell DL, O'Connor JB, et al. Pretreatment with methylprednisolone to prevent ERCP-induced pancreatitis: a randomized, multicenter, placebo-controlled clinical trial[J]. Am J Gastroenterol, 1998,93(1):61-65. doi:10.1111/j.1572-0241.1998.061_c.x.
[16] Budzyńska A, Marek T, Nowak A, et al. A prospective, randomized, placebo-controlled trial of prednisone and allopurinol in the prevention of ERCP-induced pancreatitis[J]. Endoscopy, 2001,33(9):766-772. doi:10.1055/s-2001-16520.
[17] Villar J, Ferrando C, Martínez D, et al. Dexamethasone treatment for the acute respiratory distress syndrome: a multicentre, randomised controlled trial[J]. Lancet Respir Med, 2020,8(3):267-276. doi:10.1016/S2213-2600(19)30417-5.
[18] Wang ZF, Liu C, Lu Y, et al. Dexamethasone and dextran 40 treatment of 32 patients with severe acute pancreatitis[J]. World J Gastroenterol, 2004,10(9):1333-1336. doi:10.3748/wjg.v10.i9.1333.
[19] Wan MH, Li J, Gong HL, et al. Clinical observation on the effect of dexamethasone and Chinese herbal decoction for purgation in severe acute pancreatitis patients[J]. Chin J Integr Med, 2011,17(2):141-145. doi:10.1007/s11655-011-0630-5.
